# The effect of cold application and lavender oil inhalation in cardiac surgery patients undergoing chest tube removal

**DOI:** 10.17179/excli2015-748

**Published:** 2016-01-22

**Authors:** Farzaneh Hasanzadeh, Narges Mohammadi Kashouk, Shahram Amini, Javad Asili, Seyed Ahmad Emami, Hamidreza Behnam Vashani, Amirhossein Sahebkar

**Affiliations:** 1 Department of Medical-surgical Nursing, School of Nursing and Midwifery, Mashhad University of Medical Sciences, Mashhad, Iran; 2 Department of Anesthesiology, Imam Reza Hospital, Mashhad University of Medical Sciences, Mashhad, Iran; 3 Department of Pharmacognosy, School of Pharmacy, Mashhad University of Medical Sciences, Mashhad, Iran; 4 Biotechnology Research Center, Mashhad University of Medical Sciences, Mashhad, Iran; 5 Metabolic Research Centre, Royal Perth Hospital, School of Medicine and Pharmacology, University of Western Australia, Perth, Australia

**Keywords:** chest tube, pain, anxiety, cold application, Lavandula angustifolia, nursing

## Abstract

Post-surgical chest tube removal (CTR) is associated with a significant pain and discomfort for patients. Current treatment strategies for reducing CTR-associated pain and anxiety are limited and partially efficacious. To determine the effects of cold application, inhalation of lavender essential oil, and their combination on pain and anxiety during CTR was investigated. This randomized controlled open-label trial was conducted with 80 patients in the cardiac surgery intensive care unit who had a chest tube for duration of at least 24 hours after coronary artery bypass grafting (CABG). Patients were randomized (*n*=20 in each group) to receive cold application, aromatherapy with lavender oil, cold application in combination with lavender oil inhalation, or none of the above interventions (control group). The intensity and quality of pain and anxiety were evaluated using the visual analogue scale, short form and modified-McGill pain questionnaire (SFM-MPQ) and the Spielberger situational anxiety level inventory (STAII) scale, respectively. Patients in all treatment groups had significantly lower pain intensity and anxiety compared with the control group immediately, 5, 10 and 15 min after CTR. There was no statistically significant difference in the SFM-MPQ total scores between the intervention groups. With respect to anxiety score, there was a significantly reduced anxiety level immediately after CTR in the aromatherapy and cold-aromatherapy combination groups versus the cold application group. The present results suggested the efficacy of cold application and aromatherapy with lavender oil in reducing pain and anxiety associated with post-CABG CTR.

## Abbreviations

**Abbreviations: **BMI: Body mass index; CABG: Coronary artery bypass grafting; CTR: Chest tube removal; GC: Gas chromatography; ICU: Intensive care unit; MS: Mass spectrometry; RI: Retention index; SFM-MPQ: Short form and modified-McGill pain questionnaire; STAII: Spielberger situational anxiety level inventory; VAS: Visual analogue scale

## Introduction

Insertion of chest tube following coronary artery bypass grafting (CABG) is a frequent practice aimed at maintaining hemodynamic stability and cardiopulmonary function. Chest tube facilitates drainage of the air, blood and other fluids from the pleural, pericardial or mediastinal cavity and prevents pleural effusion, pneumothorax and hemothorax. Chest tubes are typically removed 24-48 hours after surgery, or when the excess air, blood, or fluid has been properly drained. The tube adheres to the surrounding tissues, and its removal and separation of adjoined tissues is painful for the patient (Demir and Khorshid, 2010[8]). Documented sensations experienced during chest tube removal (CTR) include burning, pain, pulling, pressure, anxiety, fear and scrubbing (Houston and Jesurum, 1999[15]; Ertug and Ulker, 2012[9]). The pain experienced during CTR has been reported by patients as moderate to severe (Otaibi et al., 2013[23]). 

Pain-relieving drugs that are used for CTR-associated pain, especially opioids and NSAIDs, have partial efficacy and their use is associated with side effects such as respiratory distress, nausea, itching, and gastrointestinal bleeding. Hence, several attempts have been made to use non-pharmacological pain-relieving methods such as heat and cold therapy, hypnotism, music therapy and aromatherapy (Olapour et al., 2013[22]).

Cold applications are commonly used as a non-pharmacological method for pain relief. Cold slows down tissue metabolism and nerve conduction velocity locally and also has vasoconstrictive, anti-inflammatory, anti-spasmodic and analgesic effects. The analgesic effects of cold treatment can also be explained by the Melzack and Wall's gate control theory. According to this theory, the stimulus caused by CTR activates fibers within the parietal pleura, chest muscles, and chest tube insertion site. It is believed that activation of these pain transmitting fibers results in a release of various excitatory neurotransmitters. Application of cold may lead to a reduction or reversal of the pain impulse via activating descending inhibitory neurons that block ascending nociceptive nerves originating from the substantia gelatinosa. Reduction of anxiety is another mechanism that can lead to a decreased perception of pain (Friesner et al., 2006[11]; Demir and Khorshid, 2010[8]; Ertug and Ulker, 2012[9]).

Another non-pharmacological approach towards pain control is aromatherapy that is defined as the therapeutic use of essential oils derived from plants. Lavender oil, obtained from the plant *Lavandula angustifolia *Mill. (lavender), is a commonly used agent in aromatherapy (Sasannejad et al., 2012[24]; Bagheri-Nesami et al., 2014[4]). Lavender oil has been reported to possess several therapeutic effects including anti-anxiety, sedative, spasmolytic, antihypertensive, antimicrobial, antifungal, antiseptic and wound healing properties (Cavanagh and Wilkinson, 2002[7]; Morris, 2002[20]; Gedney et al., 2004[12]; Yip and Tse, 2006[27]; Sasannejad et al., 2012[24]; Bagheri-Nesami et al., 2014[4]). The analgesic effects of lavender oil have been widely studied (Morris, 2002[20]; Gedney et al., 2004[12]; Yip and Tse, 2006[27]; Sasannejad et al., 2012[24]). It has been shown that lavender oil can relieve the pain associated with different conditions such as labor, needle insertion, dialysis and headache (Sasannejad et al., 2012[24]; Olapour et al., 2013[22]; Bagheri-Nesami et al., 2014[4]). These effects of lavender oil are consistent with the putative efficacy of aromatherapy in reducing anxiety and fatigue (Lee, 2004[16]; Babashahi et al., 2010[3]).

Owing to the contradictory results on the efficacy of conventional pharmacological methods in relieving CTR-associated pain and discomfort, the present research was conducted to examine the effects of cold application, aromatherapy with lavender oil, and combination of both of these methods on the severity of pain and anxiety during CTR. 

## Materials and Methods

### Plant materials

Lavender oil was provided by the Giah Essence Pharmaceutical Company (Gorgan-Iran). The plant material was collected by Dr. H. Soleimani from the Tooskestan region (altitude: 400 m) located at the Southeast Gorgan, Golestan province, Iran. The plant was identified by Mrs. Souzani and a voucher sample (no. 13144) was identified and deposited in the herbarium of the School of Pharmacy, Mashhad University of Medical Sciences, Mashhad, Iran. Chemical composition of the essential oil was analyzed using Gas Chromatography (GC) and GC-Mass Spectrometry (GC-MS) methods. GC analysis was performed using a Varian CP-3800 apparatus equipped with a FID detector and fused silica column (CP-Sil 8CB, 50 m × 0.25 mm, film thickness 0.12 m). The operating conditions were oven temperature 50 °C (5 min), 50 °C-250 °C (3 °C /min), 250 °C (10 min); injector temperature 260 °C; split ratio 1:5; carrier gas N_2_ (2 ml/ min); and detector temperature 280 °C.

GC-MS analyses were performed using an Agilent 5975 apparatus with a HP-5ms column (30 m × 0.25 mm, 0.25 µm film thickness) interfaced with a quadruple mass detector and a computer equipped with Wiley 7n.l library. The operating conditions were oven temperature 50 C (5 min), 50 C-250 C (3 C/min), 250 C (10 min); injector temperature 250 °C; injection volume 0.1 µL; split ration, 1:50; carrier gas Helium at 1.1 mL/min; ionization potential 70 eV; ionization current 150 µA; ion source temperature 250 °C; and mass range 35-465 mui (Asili et al., 2009[2]).

The constituents of the oil were identified by calculation of their retention indices under temperature programmed conditions for *n*-alkanes (C8-C20) and the oil on a CP-Sil 8CB column. Identification of individual compounds was made by comparison of their mass spectra and retention indices (RI) with those of authentic samples and those reported in the literature (Adams, 200[1]7; Bozi et al. 2007[6]; Facey et al. 2005[10]). Quantification of the relative amount of the individual components was performed according to the area percentage method without consideration of the calibration factor.

### Subjects

Eighty ICU-hospitalized patients who had a chest tube for at least 24 hours after cardiothoracic surgery were included in this study. Other inclusion criteria were age > 18 yrs, body mass index (BMI) < 30 kg/ m^2^, full consciousness, ability to report pain, experiencing cardiac surgery and CTR for the first time, and having normal vital signs. Exclusion criteria were receiving mechanical ventilation support, continuous infusion of sedatives and analgesics or opioid analgesics during less than 4 hours before intervention, presence of psychiatric disease or any disease that could affect the research question (e.g. visual, auditory and smell defects), difficulty in breathing, nausea, vomiting, history of hypersensitivity or dissatisfaction during aromatherapy or cold application, and history of drug addiction or smoking. 

### Design

This study was a randomized comparative trial with a factorial design that was conducted between December 2014 and March 2015 in the cardiac surgery intensive care unit (ICU) of the Imam Reza Hospital, Mashhad, Iran. Approval for the study was obtained from the Ethics Committee of the Mashhad University of Medical Sciences (Mashhad, Iran). Written informed consent was obtained from each participant prior to the study commencement.

Eligible subjects were randomized (using random number tables) in an open-label fashion (using a random number table) to one of the following four groups:

**Group I**: applied cold with cooling gel pack (cold group, *n* = 20).

**Group II:** inhaled lavender essential oil (aroma group, *n* = 20).

**Group III:** applied cold with cooling gel pack and inhaled lavender essential oil (cold-aroma group,* n* = 20).

**Group IV:** did not receive either cold application or lavender oil inhalation (control group, *n* = 20). 

### Data collection

Pain intensity was measured using a vertical visual analog scale (VAS) from 0 to 10 (0= no pain, 10= worst pain imaginable). Numerous studies have used this scale to measure pain severity in CABG patients (Demir and Khorshid, 2010[8]; Mazloum et al., 2012[17]; Gorgi et al., 2014[14]).

The short form and modified-McGill pain questionnaire (SFM-MPQ) was used to evaluate pain quality during CTR. Reliability and validity of the MPQ was previously established by Melzack (1975[18]). The validity of SFM-MPQ in Iranian subjects was established by Mazloum et al. (2012[17]). The reliability coefficient of the tool for this study was reported to be 0.99 (Mazloum et al., 2012[17]). Validity and reliability was evaluated in our study. The Spielberger situational anxiety level inventory (STAII) was used to measure anxiety levels. The validity and reliability of this scale has been established in the Iranian population by Nazemian et al. (r= 0.89) (Nazemiyan et al., 2008[21]).

### Procedures

On the first postoperative morning, patients' readiness for CTR was determined by a physician according to the standard criteria. Patients were instructed by the researcher to use VAS, SFM-MPQ and STAII for rating pain intensity, quality and anxiety level. In addition, some information about cold application and inhalation of lavender essential oil was presented to the participants. At 6 am CTR day, an ICU nurse administered 1 gr acetaminophen intravenously to all study subjects. Cold packs were kept in freezer for at least 2 h and then were covered with gauze or special pocket (according to the manufacturer's leaflet) and applied to the area surrounding the chest tube until the skin temperature reached 13 °C.

The researcher asked the patients to mark the pain intensity, quality and situational anxiety they felt with the chest tube in place. Pain intensity was measured 10 minutes before, and immediately, 5, 10, and 15 minutes after CTR. Pain quality and situational anxiety of the patients were measured 10 minutes before and immediately after CTR.

All patients were reassured that analgesic drugs would be administered as needed. All chest tubes were removed by the same ICU nurse to ensure the reliability of the results. The procedures for the application of cold and aromatherapy are as follows.

### Cold application

In the cold application group, cooling gel packs (Dispotch Company, Italy) were used to reduce the body temperature around the chest tube during undressing. The bandage was removed from the chest area and body temperature was measured and recorded by a non-contact infrared thermometer (UNI-T 912, Hong Kong). Then, a single layer of sterile gauze pad was placed around the skin area of pericardial tube insertion (according to the regulations of the Imam Reza cardiac surgery ICU, pericardial tube is the first tube that is removed) and cooling packs (14×18) were twisted in gauze and placed on top of it.

Cold application was terminated when the skin temperature reached 13 °C. After removal of cooling packs, the nurse removed the pericardial tube within 1-2 minutes. The intensity and quality of pain experienced by the patient and the anxiety level were measured and recorded at the time points described above. 

### Aromatherapy

In the aromatherapy group, 1-2 drops of aromatherapy blend containing lavender essential oil were poured on cotton and fixed at a 10 cm distance from the patients' nose. The patient was asked to breathe slowly for 20 minutes and then pain and anxiety were measured as described above.

### Statistical analysis

SPSS software version 11.5 was used to analyze the recoded data. Descriptive statistics were used to summarize demographic characteristics of the study participants. Normality of data were checked using Kolmogorov-Smirnov and Shapiro-Wilk tests. Baseline parameters of the study groups were compared using one-way ANOVA, Chi-square and Fisher's exact tests were used. To compare the efficacy measures among the study groups, one-way ANOVA, Kruskal-Wallis, and repeated measures ANOVA tests were used. Bivariate correlations between anxiety and pain scores before and immediately after CTR were performed using Pearson's correlation coefficient. In all analyses, a two-sided P value of <0.05 was considered as statistically significant.

## Results

### Essential oil composition

Ninety-three compounds were identified in the lavender oil, representing 99.0 % of the total oil composition (Table 1(Tab. 1)). The main volatile constituents were linalool (21.1 %), 1,8-cineol (15.7 %), and borneol (11. 6 %). The oil was predominated by oxygenated monoterpenes (81.3 %), followed by monoterpene hydrocarbons (7.2 %), oxygenated sesquiterpenes (4.8 %) and sesquiterpene hydrocarbons (3.6 %).

### Baseline and demographic characteristics

The mean age of participants was 54.3 ± 10.9 yrs. The study population consisted of 56.2 % men and 43.8 % women, and distribution of genders among the study groups was similar (P=0.77). All subjects had two chest tubes (pericardial and left pleural) after CABG.

Mean tube remaining time was 45.9±1.3 hrs, and there was no significant difference among the study groups regarding this parameter (P=0.17). Likewise, the groups were comparable with respect to demographic characteristics including marital status (P=0.23), education (P=0.86), and residential status (urban or rural) (P=0.91), the worst pain experienced in the past (P=0.58) and its description (P=0.33). Baseline characteristics of the study groups are summarized in Table 2(Tab. 2). 

### Pain intensity

**Figure 1:**
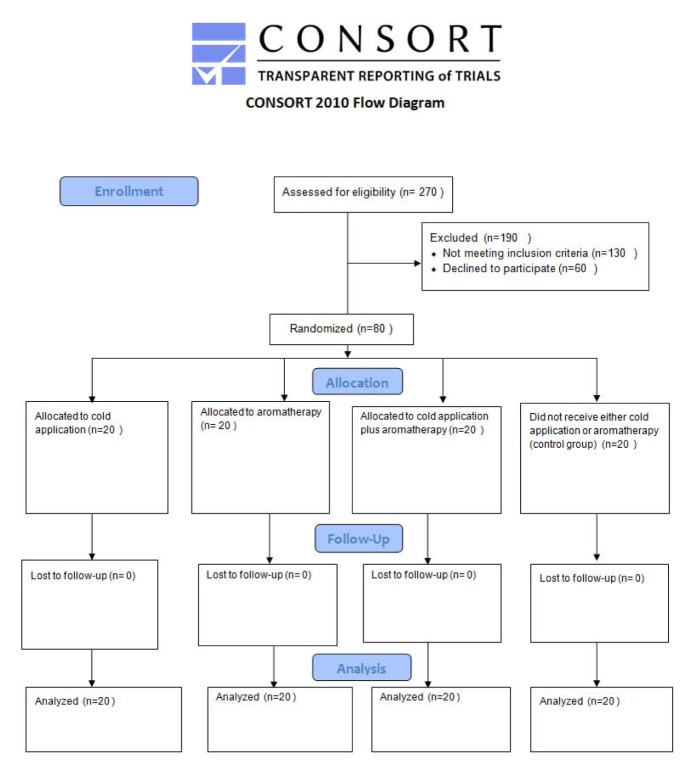
Flow diagram of the study

**Figure 2:**
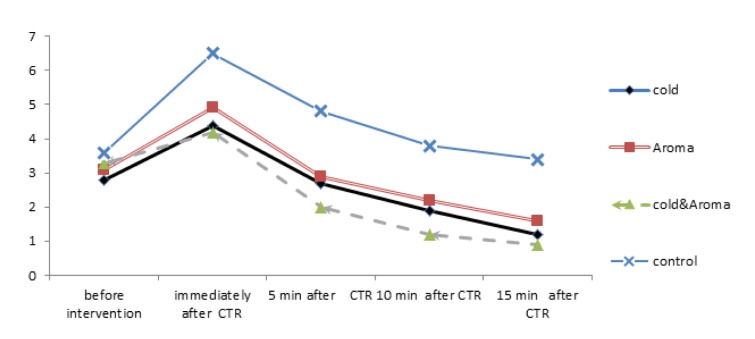
Trend of changes in pain intensity in the study groups at different time points

The study flow diagram is show in Figure 1(Fig. 1). The pain intensity scores of the 4 study groups in 5 time points (before, immediately, 5, 10 and 15 min after CTR) are shown in Figure 2(Fig. 2) and Table 3(Tab. 3). There was no significant difference in the baseline pain intensity among the study groups (*P=0.55*). Repeated-measures analysis revealed a significant increase in pain severity immediately after CTR, followed by a continuous decreasing trend until the last assessed time point in all study groups (*P<0.001*). *Post-hoc* multiple comparisons revealed significantly reduced pain intensity in all intervention groups compared with the control group immediately after CTR, as well as at 5, 10 and 15 min post-CTR time points. However, there was no significant difference among cold application, aromatherapy and cold-aroma groups.

### Pain quality

The words selected by most of the subjects to describe the present pain endured during CTR were “discomforting” (reported by 37.5 %), “distressing” (22.5 %), and “horrible” (21.2 %). Also, the words selected by subjects to describe pain during CTR, according to the SF-MPQ sclae were “fearful” (70 %), “shooting” (51.2 %), “tender” (51.2 %), “sickening” (48.8 %), “hot or burning” (46.2 %) and “sharp” (30 %). The total SFM-MPQ score in cold, aroma, cold-aroma and control groups were 19.5±5.8, 21.9±7.2, 17.1±9.9 and 29.1±5.5, respectively. All interventions were associated with a reduced total SFM-MPQ score compared with the control group (*P<0.001* [cold and cold-aroma groups] and *P=0.020* [aroma group]); however, there was no statistically significant difference between the interventions.

### Anxiety

Table 4(Tab. 4) represents the level of anxiety according to the STAII scale before and immediately after CTR. Anxiety score decreased in all study groups (*P<0.001*). *Post-hoc* multiple comparisons revealed a significant difference between the aroma group with cold (*P<0.009*) and control groups (*P<0.007*), and also between the cold-aroma group with cold (*P<0.001*) and control groups (*P<0.001*).

### Bivariate correlation analysis

Bivariate correlations using Pearson's correlation coefficients did not suggest any significant correlation between anxiety and pain scores either before or immediately after CTR. Likewise, there was no significant correlation between age, sleep duration, length of chest tube in the body, duration of chest tube presence in the body and fatigue score with either pain or anxiety score immediately after CTR.

## Discussion

Findings of the present study showed that all three types of interventions (cold application, inhalation of lavender essential oil, and combination of cold and lavender essential oil inhalation) could reduce pain and anxiety associated with CTR. However, there was no significant difference among the applied interventions, and combination therapy did not further improve the analgesic and anti-anxiety effects of each intervention. Although numerous studies have shown the efficacy of non-pharmacological methods in reducing pain, to the best of authors' knowledge, this is the first study comparing the effects of cold application and lavender aromatherapy in reducing the pain associated with CTR. In order to eliminate the confounding effect of surgery type and chest tube type on the study outcomes, CTR-associated pain was assessed in subjects undergoing pericardial tube removal following CABG. 

The composition of lavender oil that was used in this study was predominated by oxygenated monoterpenes. This result is consistent with previous phytochemical reports on the same plant. Barazandeh et al. (1999[5]) studied the composition of the oil obtained from the aerial parts of *L. angustifolia.* The volatile oil of the herb had 19 compounds representing 90.4 % of the oil composition, and the main components were linalool (39.5 %), terpinen-4-ol (7.3 %) and linalyl acetate (32.4 %). Recently, Tayarani-Najaran et al. (2014[26]) identified 80 compounds representing 97.4 % of the total lavender oil composition. The main compounds were reported to be 1, 8-cineol (18.8 %) and borneol (19. 6 %).

In previous studies, lavender oil was shown to be useful in reducing the pain associated with migraine, cesarean section, and needle insertion into a fistula in hemodialysis patients (Sasannejad et al., 2012[24]; Olapour et al., 2013[22]; Bagheri-Nesami et al., 2014[4]). There have been also some reports on the use of cold application for relieving the pain after CTR (Demir and Khorshid, 2010[8]; Mazloum et al., 2012[17]; Otaibi et al., 2013[23]; Gorgi et al., 2014[14]). Gorgi et al. (2014[14]) studied the effects of relaxation and cold application on the pain severity following cardiac surgery and reported a significant effect of both interventions versus control. Similar to the present study, the authors did not find any difference between the analgesic effects of cold application and relaxation; however, the impact of combination therapy with both methods was not investigated. 

Pain is a multidimensional sensation which is perceived in each subject differently. It includes a blend of neurophysiologic, biochemical, cultural, cognitive, and environmental dimensions (Sauls, 2002[25]). In this study, we assessed all 11 quality descriptors of the SFM-MPQ scale to describe the quality of pain experienced after CTR, thus enabling the participants to quantify and describe both sensory and affective components of their pain experience.

The MPQ words selected most frequently by subjects in this study were “fearful”, “shooting”, “tender”, “terrible” and “stinging”. In earlier studies on CTR, the frequency of selection of “terrible” from the MPQ varied considerably. Sauls (2002[25]) found these words were chosen less often than another affective descriptor, “cramping” and “punishing-cruel”. In the study by Demir and Khorshid, “stinging” (86.7 %) and “sore” (74.4 %) were reported as the predominant descriptors of pain experienced during CTR (Demir and Khorshid, 2010[8]).

In the literature, there has been only a few studies related to anxiety associated with CTR. Mimnaugh et al. (1999[19]) reported that anxiety levels were high in 77 % of patients during CTR. Gift et al. (1991[13]) also reported that the CTR procedure induces anxiety. In the Demir study, patients experienced high anxiety levels before CTR and moderate anxiety immediately after CTR, and there was no statistical difference in the variation of anxiety of patients among the groups receiving cold application, relaxation and control treatment (Demir and Khorshid, 2010[8]). In the present study, anxiety scores were decreased in all groups immediately after CTR. The highest decrease was observed in the groups receiving aroma and cold-aroma combination. Overall, patients' anxiety was improved by interventions compared with the control group; however, a moderate anxiety level was still present immediately after CTR.

As a limitation, this study was performed on a relatively small number of subjects and was limited to patients who underwent CABG surgery. Whilst this inclusion criteria increase the homogeneity of the study population, it will make generalizability of findings difficult. Moreover, the subjective nature of efficacy measures might have introduced bias into the results, though the effect of such a potential bias could be minimized by the randomized design of the study. Finally, this study was performed in an open-label fashion because the nature of interventions made it impossible to design appropriate placebo.

In conclusion, the findings of the present randomized controlled trial suggested that cold application, aromatherapy, and cold application in combination with lavender oil inhalation could be used as non-pharmacologic interventions to relieve pain associated with CTR. Given the simplicity and inexpensive nature of such approaches, their application in clinical practice could be suggested to the nursing staff. However, before routine application, confirmation of the present findings in larger scale trials, and in CTR-associated pain of non-CABG subjects is recommended. 

## Acknowledgements

This study was financially supported by the Research Council at the Mashhad University of Medical Sciences, Mashhad, Iran.

## Conflict of interest

The authors have no competing interests to disclose.

## Figures and Tables

**Table 1:**
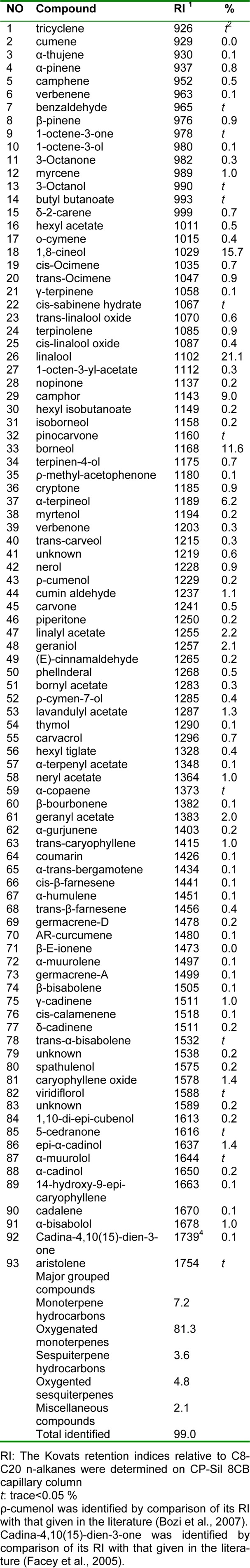
Essential oil components of the aerial parts of *Lavandula angustifolia*

**Table 2:**
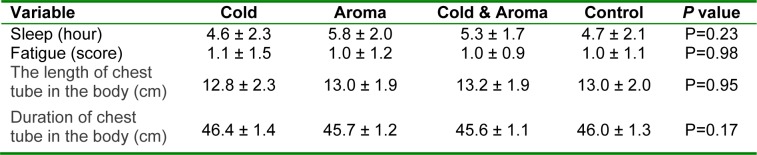
Comparison of baseline characteristics among the study groups

**Table 3:**
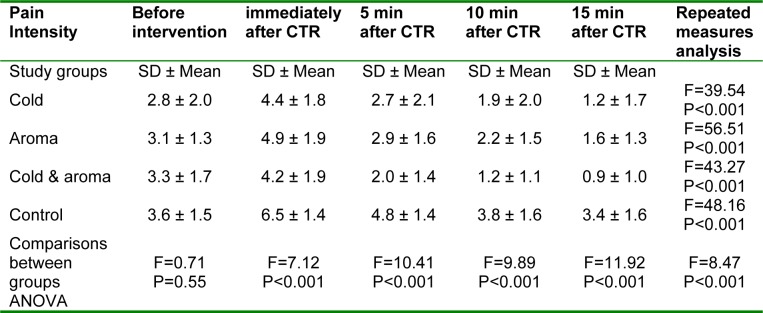
Comparison of pain intensity scores among the study groups at different time points

**Table 4:**
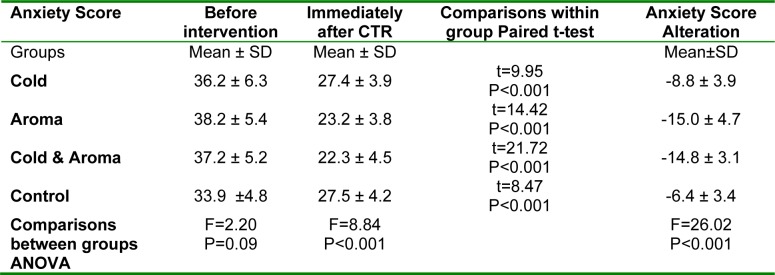
Comparison of anxiety scores among different study groups

## References

[1] Adams RP (2007). Identification of essential oil components by gas chromatography/quadrupole mass spectroscopy.

[2] Asili J, Sahebkar A, Fazly Bazzaz BS, Sharifi S, Iranshahi M (2009). Identification of essential oil components of Ferula badrakema fruits by GC-MS and13C-NMR methods and evaluation of its antimicrobial activity. J Essent Oil-Bear Plants.

[3] Babashahi M, Fayazi S, Aghel N, Haghighizadeh MH (2010). Effect of aromatherapy on anxiety level among preoperative patients. Sci Med J.

[4] Bagheri-Nesami M, Espahbodi F, Nikkhah F, Shorofi SA, Yazdani J (2014). The effects of lavender aromatherapy on pain following needle insertion into fistula in hemodialysis patient. Complement Ther Clin Pract.

[5] Barazandeh MM (1999). Essential oil composition of two Lavandula species from Iran and France by capillary gas chromatography method. (in Persian). Iran Med Aromat Plants Res.

[6] Bozi J, Czagany Z, Meszaros E, Blazso M (2007). Thermal decomposition of flame retarded polycarbonates. J Anal Appl Pyrolysis.

[7] Cavanagh HMA, Wilkinson JM (2002). Biological activities of lavender essential oil. Phytother Res.

[8] Demir Y, Khorshid L (2010). The effect of cold application in combination with standard analgesic administration on pain and anxiety during chest tube removal: A single-blinded, randomized, double-controlled study. Pain Manag Nurs.

[9] Ertug N, Ulker S (2012). The effect of cold application on pain due to chest tube removal. J Clin Nurs.

[10] Facey PC, Porter RBR, Reese PB, Williams LAD (2005). Biological activity and chemical composition of the essential oil from Jamaican Hyptis verticillata Jacq. J Agric Food Chem.

[11] Friesner SA, Curry DM, Moddeman GR (2006). Comparison of two pain management strategies during chest tube removal: relaxation exercise with opioids and opioids alone. Heart Lung.

[12] Gedney JJ, Glover TL, Fillingim RB (2004). Sensory and affective pain discrimination after inhalation of essential oils. Psychosom Med.

[13] Gift AG, Bolgiano CS, Cunningham J (1991). Sensetions during chest tube removal. Heart Lung.

[14] Gorgi HM, Nesami BM, Ayyasi M, Ghafari R, Yazdani J (2014). Comparison of ice packs application and relaxation therapy in pain reduction during chest tube removal following cardiac surgery. North Am J Med Sci.

[15] Houston S, Jesurum J (1999). The quick relaxation technique: Effect on pain associated with chest tube removal. Appl Nurs Res.

[16] Lee SH (2004). Effects of aroma inhalation on fatigue and sleep quality of postpartum mothers. Korean J Women Health Nurs.

[17] Mazloum SR, Abbasi Tehnizi M, Kiannejad A, Gandomkar F (2012). Effect of applying ice bag on pain intensity associated with chest tube removal after cardiac surgery. Horizon Med Sci.

[18] Melzack R (1975). Prolonged relief of pain by brief, intense transcutaneous somatic stimulation. Pain Manag Nurs.

[19] Mimnaugh L, Winegar M, Mabrey Y, Davis JE (1999). Sensations experienced during removal of tubes in acute postoperative patients. Appl Nurs Res.

[20] Morris N (2002). The effects of lavender (Lavendula angustifolium) baths on psychological well-being: two exploratory randomized control trials. Complement Ther Med.

[21] Nazemiyan F, Ghafari F, Pourghaznein T (2008). Study of depression and anxiety in hemodialysis patients. Med J Mashhad Uni Med Sci.

[22] Olapour A, Behaeen K, Akhondzadeh R, Soltani F, Razavi F, Bekhradi R (2013). The effect of inhalation of aromatherapy blend containing lavender essential oil on cesarean postoperative pain. Anesth Pain.

[23] Otaibi R, Mokabel F, Ghuneimy Y (2013). The effect of cold application on pain and anxiety during chest tube removal. J Am Sci.

[24] Sasannejad P, Saeedi M, Shoeibi A, Gorgi A, Abbasi M, Foroughipour M (2012). Lavender essential oil in the treatment of migraine headache: A placebo-controlled clinical trial. Eur Neurol.

[25] Sauls J (2002). The use of ice for pain associated with chest tube removal. Pain Manag Nurs.

[26] Tayarani-Najaran Z, Amiri A, Karimi G, Emami SA, Asili J, Mousavi SH (2014). Comparative studies of cytotoxic and apoptotic properties of different extracts and the essential oil of lavandula angustifolia on malignant and normal cells. Nutr Cancer.

[27] Yip YB, Tse SH (2006). An experimental study on the effectiveness of acupressure with aromatic lavender essential oil for sub-acute, nonspecific neck pain in Hong Kong. Complement Ther Clin Pract.

